# Externalizing Behaviors Buffer the Effects of Early Life Adversity on Physiologic Dysregulation

**DOI:** 10.1038/s41598-019-49461-x

**Published:** 2019-09-20

**Authors:** Stacey N. Doan, Nadya Dich, Thomas E. Fuller-Rowell, Gary W. Evans

**Affiliations:** 1 850 Columbia Ave, Department of Psychological Science, Claremont McKenna College, Claremont, CA 91711 USA; 2 Department of Public Health, University of Copenhagen, Copenhagen, Denmark; 3 College of Human Sciences, Auburn University, Auburn, AL USA; 4 Design and Environmental Analysis, Department of Human Development, Cornell University, Ithaca, USA

**Keywords:** Paediatric research, Risk factors

## Abstract

The present study examined the counterintuitive hypothesis that externalizing behaviors such as aggression, although in many respects detrimental, may be functional and protect against the detrimental health consequences of early life adversity. In particular, in line with evolutionary models of development, we argue that externalizing problems moderate the association between chronic stress exposure and allostatic load, a biological marker of chronic physiological dysregulation. Prospective interactive effects of externalizing behaviors and cumulative risk (a confluence of multiple risk factors) on children’s allostatic load were assessed in 260 children (46% female, baseline age = 9). Exposure to early life adversity was assessed at baseline using a cumulative risk index. Externalizing behaviors were reported by parents at baseline. Allostatic load was measured at baseline and at ages 13 and 17, using endocrine, cardiovascular and metabolic parameters. Results of linear-mixed effects models indicated that the association between cumulative risk and allostatic load was attenuated for adolescents who scored high on externalizing behaviors. Further examination of sex differences indicated that the findings were more pronounced among males than females.

## Introduction

Traditional developmental pathology models have operated under the assumption that early life adversities lead to maladaptive behaviors^1–3^. In particular, externalizing behaviors^4^ have been considered maladaptive because they are associated with a variety of negative outcomes such as underachievement^2^, peer rejection^5^ and substance use^6^. However, recent theoretical perspectives have argued that the presumption of externalizing behaviors being pathological is problematic and limits our understanding of effective interventions^7^. In particular, the specialization and sensitization hypothesis suggests that adversity does not simply lead to deficits, but also shapes behaviors to maximize fitness and adaptation to the developmental context^8^. From this perspective, problematic behaviors, including externalizing behaviors, are not “deficits” but rather an adaptive response to stressful, unstable or otherwise disadvantageous environments. Thus, for interventions to be effective they must consider the possibility that these behaviors serve important *functions*. In the proposed study, we test the counterintuitive hypothesis that, in an ecological context of high risk exposure, externalizing behaviors are beneficial for physical health.

## Rethinking the Functions of Externalizing Behaviors

In contrast to traditional models of developmental psychopathology, which argue that externalizing behaviors are a pathological/undesirable consequence of adversity, the evolutionary developmental approach posits that externalizing behaviors are more accurately considered adaptive adjustments to stressful, risky environments^7^. Adaptive traits are those that have evolved through natural selection due to their effects on survival and reproductive success^9^. To enhance survival within specific contexts, neurobiological mechanisms function to calibrate developmental and behavioral strategies to match the ecological context wherein they operate^10–12^. This view of development challenges the prevailing psychopathology analysis of dysfunctional outcomes within settings of adversity. The evolutionary perspective contends that both stressful and supportive environments have been part of the human experience throughout our history, and that developmental systems shaped by natural selection respond adaptively to both kinds of contexts^13^. Thus, stressful environments do not so much disturb development as direct or regulate it toward strategies that are adaptive under stressful conditions (or at least were adaptive during human evolutionary history). This is not to say that behaviors such as aggression or stealing are acceptable or good solutions for dealing with high risk environments in contemporary times. Our point is rather that although high levels of externalizing are harmful both to individuals and to those around them, in particular with respect to social functioning, they may at the same time offer some modicum of protection against some of the other outcomes of risky settings. In particular, externalizing behaviors may partially protect youth from the harmful impacts of elevated allostatic load (an index of chronic physiological stress) caused by repeated mobilizations of the body’s stress response systems^14^.

The relationship between the stress response system and aggression has been documented in both animals and humans. In the animal literature, researchers have argued that aggression can be a stress-reducing behavioral outlet^15^. For example, multiple rat studies have demonstrated that biting attenuates physiological stress responses by suppressing stress-induced dopamine metabolism in the striatum^16^, turnover of catecholamines in the central nervous system^17^, as well as suppression of corticotrophin-releasing factor in the hypothalamus^18^. In non- human primate models, a relationship between aggression and the physiological stress response has also been found^19^. In both dominant^20^ and subordinate^21^ male baboons, aggression was related to lower basal cortisol concentrations. Male undergraduates who were allowed to shock another individual after a frustration sequence exhibited decreased diastolic blood pressure^22^. Externalizing behaviors have also been found to be associated with lower salivary cortisol in boys^23^, echoing the expansive literature demonstrating that aggressive behavior after a stressor decreases glucocorticoid secretion.

In addition to potential direct effects on the body’s stress physiology, externalizing behaviors such as aggression and stealing under certain circumstances can function to control and increase access to physical, social and sexual resources^24,25^. To illustrate, stealing can allow one to have access to material goods that may be impossible or difficult otherwise. In discussing the evolutionary basis of risky adolescent behavior, Ellis and colleagues emphasized that adolescence is an important inflection point, whereby status, resource control and mating success are particularly salient^7^. Moreover, certain strategies such as aggression and bullying can be adaptive in risky contexts by conferring and elevating social status^26^. Consistent with this perspective, bullying predicted dating behavior in adolescence, such that bullying behavior increases sexual opportunities^25^. In sum, by increasing access to these material and social resources, externalizing behaviors can mitigate the impact of external stressors on physiological functioning.

Despite both theoretical and empirical research supportive of the hypothesis that externalizing behaviors may be an effective physiological regulation mechanism, to the best of our knowledge, no study has investigated the extent to which externalizing behaviors can buffer against the effect of environmental stressors on human physiology. In the current study, we use a longitudinal prospective design to test the hypothesis that externalizing behaviors will mitigate the effects of high adversity (operationalized as cumulative risk (CR) exposure), leading to less stress-induced arousal in the HPA axis and interconnected physiological systems.

## Cumulative Risk as a Developmental Context

In assessing exposure to adversity, researchers often focus on one variable such as family income or child abuse. However, building upon the bioecological model of human development^27^, cumulative risk approaches to studying adversity argue that children’s developmental outcomes are better predicted by examining the *accumulation* of risk factors. The number of risk factors present is a better predictor of children’s health and well-being than any one risk factor or subset thereof  ^28–31^. In addition to the number of risk factors, timing also matters. A growing body of research has demonstrated that adversity is particularly detrimental when experienced during the childhood years (prior to adolescence). Indeed, early life adversity predicts a wide range of physical^32^ and mental health outcomes^33,34^. The influence of early life adversity is both broad and severe^35–37^ with early adverse life events leading to increased incidence of mental and physical health disorders in adolescence and adulthood^38^. Finally, there is also an abundance of research demonstrating that the stress response system is particularly susceptible to outside influences during the early years^39^. Thus, in the current study, rather than looking at any single indicator of adversity (e.g., income, education), we take a cumulative risk approach and measure a broad range of social and physical risks^28,35,40^. With regards to timing, we focus on exposure during the early childhood years (before age 9, prior to adolescence) as research suggests that early childhood is a time of vulnerability and plasticity with regards to the stress response system.

## Allostatic Load as Indicator of Chronic Physiological Stress

In the current study, we use allostatic load as an outcome, and our measure for chronic physiological stress. The allostatic load model, developed by Bruce McEwen and colleagues, posits that the stress response is multifaceted. The secretion of catecholamines and glucocorticoids affect the functioning of multiple brain systems and interconnected physiological systems. In particular, stress influences the cardiovascular, metabolic and immune systems. Allostatic load (AL) then refers to the cumulative effects of chronic stress exposure on the body. AL has often been described as an index of “wear and tear” on the body resulting from a chronic hyperactivity of the stress response system. AL has been thoroughly investigated in adults, with data showing that it is linked with poor physical health^41,42^, inversely related with socio-economic status^43^, and positively associated with levels of neighborhood poverty^44^ and early childhood adversity^45^.

In terms of measurement, the AL model emphasizes the necessity of measuring multiple biomarkers across metabolic, endocrine, and immune systems^42^. This is in contrast to approaches that often only focus on one indicator (e.g., cortisol). Conceptually, because stressors in the environment activate multiple, interconnected systems, measuring multiple indicators is thought to better capture the influence of stress on the body’s physiological systems. These multiple indicators may include both primary mediators in the cascade from stress to health, and secondary mediators that reflect, for example, metabolic functioning. Summary indicators that capture a measure of biological dysregulation across multiple systems are both better predictors and more accurate warnings of later health outcomes^42^.

## Possible Sex Differences

In many cultures there are significant social sanctions disouraging girls to be aggressive, whereas being a “man” also involves at least a modicum of displays of externalizing behaviors^46^. Consistent with this idea, biosocial perspectives argue that sex differences in physical attributes make it less desirable for women to engage in aggressive behaviors^47^. Moreover, social norms tend to reward men for fighting and punishing females^48^. Finally, evidence suggests that aggressive women are evaluated more negatively than aggressive men^49^ and that female aggressors are sanctioned more significantly than male aggressors^50^. In line with these ideas, research suggests that sex differences in externalizing behaviors^51^ are robust, and likely to be driven by sexual selection^52^. Moreover, research on stress regulation suggests that “fight-or-flight” tendencies in the face of adversity are more common for males^53^. Rather than “fight-or-flight” responses, the female dominant coping mechanism is to“tend-and-befriend”^53^. Therefore we expect that the protective effects of externalizing behaviors will be more pronounced for boys.

In the current study, we use a prospective longitudinal design to test a counterintutive hypothesis that externalizing behaviors such as aggression and stealing can serve an adaptive function for children growing up in high risk contexts. Specifically, we argue that externalizing behaviors will buffer the effects of cumulative risk exposure on allostatic load, a physiological indicator of cumulative stress. Moreover, we expect that the health protective effects of externalizing behaviors in the context of chronic stress exposure will be more pronounced among boys.

## Methods

### Participants

Data from the current study were collected at three waves. At the first wave of data collection 341 adolescents and their parents (174 males, mean age 9.2, *SD* = 1.2) participated. Participants came from rural upstate New York. Low-income families were over-sampled (income to needs ratio ≤1 for 37% of families). Participants participated again four years later at Wave 2 (mean age = 13.4, *SD* = 1.0, and at eight years from baseline at Wave 3 (mean age = 17.3, *SD* = 1.0). Participants who did not provide AL data for any of the three waves (*N* = 64) were excluded. An additional 17 participants were excluded due to missing information on one or more of the covariates. The final sample for the study included 260 children (118 female). The excluded participants had higher CR scores, as well as higher levels of externalizing behaviors (p-values for the between-group differences = 0.01 and <0.001 respectively). Females were more likely to be excluded than males (p-values for the between-group differences = 0.01). The study was approved by Cornell University’s Institutional Review Board. Parents provided informed consent and youth provided assent. All methods were performed in accordance with the relevant guidelines and regulations.

### Measures

#### Cumulative risk

Cumulative risk was assessed at Wave 1 (mean age 9.2 years). In the current study, we assessed cumulative risk using a summary measure capturing nine risk factors from three domains: demographic, psychosocial, and environmental. Demographic (maternal high school drop-out, single parent, and household income at or below the poverty line) and psychosocial (family separation, family turmoil, exposure to violence) factors were reported by mothers during an interview at their homes. Environmental factors (residential density, noise levels, housing quality) were assessed by trained raters during home visits. For a more detailed explication of how each of the risk factors was measured, see our past publications^40,54^. As critically reviewed in Evans *et al*.^28^ and as recommended therein, we employ an additive model, dichotomizing each continuous risk factor at the upper quartile and using standard risk definitions for categorical variables. The continuous variables herein include child separation from family, family turmoil, exposure to violence, residential density, noise levels, and housing quality. Scores on the top quartile of these continuous measures received a 1 for each category. Additionally, three categorical risks (maternal high school drop-out, single parent, and household income at or below the poverty line) were included, yielding a possible CR range 0–9.In Table 1, we report the descriptives for our cumulative risk variable. For a detailed breakdown of each individual risk factor, we refer the reader to our prior publication^40^ or the supplementary materials.

**Table 1:** Means, observed ranges, and standard deviations of allostatic load at Waves 1–3, cumulative risk at Wave 1 and Externalizing behavior at Wave 1 and zero-order correlations among the variables.

		N	Observed Range	Mean	SD	2.	3.	4.	5.
1.	Allostatic Load W1	235	0–4	1.05	0.99	0.36^**^	0.19^*^	0.14^*^	0.00
2.	Allostatic Load W2	163	0–5	1.37	1.16		0.19^*^	0.06	0.00
3.	Allostatic Load W3	178	0–4	1.24	1.07			0.21^**^	0.13
4.	Cumulative Risk	260	0–7	1.80	1.65				0.31^**^
5.	Externalizing behavior	260	0–6	0.96	1.26				

Note: *p < 0.05, **p < 0.01.

#### Externalizing behaviors

Questions assessing externalizing behaviors were drawn from the well-established standardized Rutter Child Behavior Questionnaire (Rutter, Tizard & Whitmore, 1970). Parents reported on children’s externalizing behaviors at Wave 1. Four questions, rated on a three-point scale, asked about the following behaviors: stealing, destroying other’s property, disobedience and bullying behavior. The reliability coefficient for this scale was Cronbach’s alpha = 0.74.

#### Allostatic Load

We measured allostatic load at all three waves. AL included six parameters capturing the activity of the HPA axis (12 hour, overnight urinary cortisol), SAM system (12 hour urinary epinephrine and norepinephrine), cardiovascular system (resting systolic and diastolic blood pressure), and metabolic system (body mass index). Urine samples were stored with a preservative (i.e., metabisulfite). We recorded total volume, extracted duplicate 10-mL samples, and acidified the catcholamine aliquots, which were then were deep-frozen (−80 °C) until assays were completed. Epinephrine and norepinephrine were assayed by HPCL with electrochemical detection^55^, and cortisol was assayed with a radioimmune assay^56^. Additionally, creatinine was included as a statistical control for the neuroendocrine assays. Blood pressure was assessed with automated readings (Dinamap Model Pro 100, Critikon) taken every two minutes while participants sat quietly. We averaged the second through seventh readings and used the mean as the index of resting blood pressure^57^. Finally, body mass index was calculated as weight in kilograms divided by squared height in meters.

Consistent with past work, AL score was calculated as the number of physiological parameters on which participants scored above the high-risk cut-off (possible range 0 to 6). We used clinical cut-offs for blood pressure (BP) and body mass index (BMI). BP and BMI percentiles were calculated based on age-adjusted pediatric norms, and BP norms were adjusted for height^58,59^. Because there are no established clinical cut-offs for neuroendocrine biomarkers, we used 75^th^ percentile cut-off thresholds for cortisol and catecholamines.

### Statistical analyses

The repeated-measures data were analyzed using linear mixed-effect (LME) models^60^ with participants’ intercepts as random effects. In this model, AL was the outcome and CR, externalizing, and gender were the predictors. This approach allowed us to account for the interdependence of observations from the same child and, at the same time, to include participants with missing AL data for one or two waves, as missing data estimation is an integrated part of LME model estimation procedure. To test the principal hypothesis of the study that externalizing behaviors buffer the effects of CR on AL and that this buffering effect varies by gender, we fit a 3-way interaction model with CR, externalizing, gender, and their 3-way interaction terms entered as time-invariant fixed effects. The analyses also controlled for age (time-varying fixed effect), race [White, Nonwhite], and emotional temperament [measured at baseline using the Emotionality subscale of the Buss and Plomin’s^57^ Emotionality, Activity and Sociability (EAS) Survey] (time-invariant fixed effects). We controlled for race because race differences in allostatic load have been demonstrated in past research^61^. Moreover, because emotionality has been found to be associated with behavioral problems^62^ and increased stress reactivity^63^, we controlled for emotionality in order to rule out the effect of this personality characteristic. In order to plot the three-way interaction, we calculated the slopes of CR at low (0, no externalizing symptoms) and high (one standard deviation above the mean) levels of externalizing in males and females. Deidentified data from the current study can be accessed by contacting the corresponding author.

## Results

Descriptive data on study variables and their zero order correlations are depicted in Table 1. CR was correlated with AL at Wave 1 and Wave 3 and with externalizing behavior. Externalizing was not correlated with AL at any of the three waves. All measures of AL were inter-correlated.

### Buffering effects of externalizing behavior on cumulative risk

**Figure 1:**
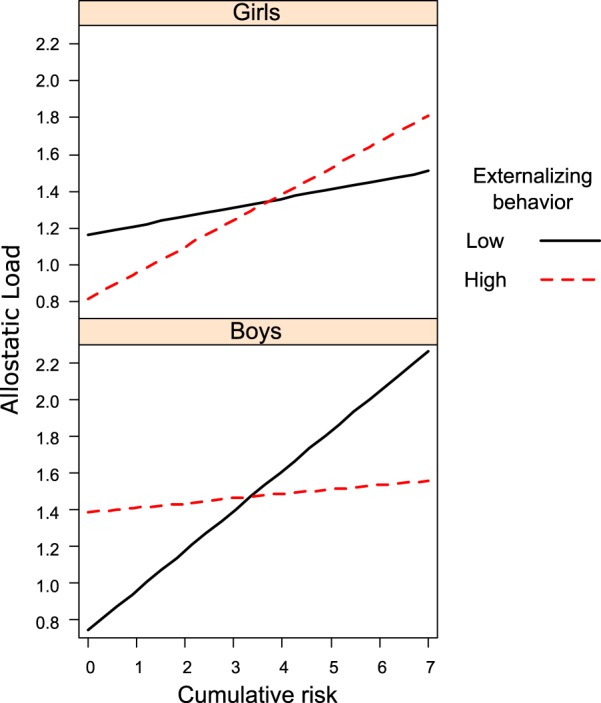
The effects of cumulative risk exposure on allostatic load depend on gender and externalizing behaviors.

Table 2 presents coefficients and standard errors for the three-way interaction model testing the buffering effects of externalizing behavior on the association between cumulative risk and AL by gender. As shown in Fig. 1, the three-way interaction between gender, externalizing behavior and CR was statistically significant (*p* = 0.030), suggesting that the moderating effects of externalizing behaviors depended on gender. In males, the interaction between CR and externalizing behavior was significant (*p* = 0.011). When no externalizing symptoms were present, the slope of CR was 0.22 (*SE* = 0.06, *p* < 0.001). However, when externalizing symptoms were high (1 standard deviation above the mean), the effect of CR on AL was not statistically significant (*b* = 0.05, *SE* = 0.06, *p* = 0.39). In females, the interaction between CR and externalizing was not statistically significant (*p* = 0.39). The slope of CR tended to increase with increasing levels of externalizing in females. However, the slope for CR and AL was only marginally significant at levels of externalizing one SD above the mean (*b* = 0.13, *SE* = 0.07, *p* = 0.067) and not significant at low levels of externalizing behaviors (*b* = 0.05, *SE* = 0.06, *p* = 0.42).

**Table 2:** Fixed effect coefficients and standard errors for the Linear Mixed Effect interaction model testing gender differences in the buffering effects of externalizing behavior on the association between cumulative risk and allostatic load.

	b	SE	p
Age in years	0.02	0.01	0.077
Race [Nonwhite]	−0.04	0.19	0.85
Negative Emotionality (range 1–5)	−0.06	0.06	0.31
Gender [female]	0.42	0.19	0.024
CR	0.22	0.06	<0.001
EXT	0.26	0.10	0.010
Gender [Female] × CR	−0.17	0.08	0.049
Gender [Female] × EXT	−0.40	0.16	0.015
CR × EXT	−0.08	0.03	0.011
Gender [Female] × CR × EXT	0.11	0.05	0.030

Note: CR = Cumulative Risk, EXT = externalizing behavior.

## Discussion

This is the first study that investigates the moderating role of externalizing behaviors in the association between early life adversity and allostatic load. We hypothesized that for youth growing up in the context of adversity, externalizing behaviors would buffer the detrimental effects of cumulative risk on physiological dysregulation/allostatic load. In other words, we reasoned that behaviors such as aggression may be an effective stress regulation strategy under conditions of high-risk exposure. Additionally, we hypothesized that due to gendered social norms surrounding aggression, the protective effect would be most pronounced among males. Results were consistent with both our hypotheses. As expected, there was a main effect of cumulative risk on allostatic load, but this relationship was attenuated among youth who exhibited higher levels of externalizing behaviors. This moderation effect was present for males, but not females.

The interaction pattern between externalizing and cumulative risk exposure is consistent with evolutionary models of psychopathology hypothesizing that behaviors which are often considered maladaptive can actually, in some circumstances, be adaptive^7,64^. Consistent with these models, there is evidence to suggest that some aggressive adolescents can become dominant, respected and popular^65,66^. We, however, suggest an additional, and perhaps just as primary, function of externalizing behaviors. Consistent with animal studies demonstrating that aggression may act as a safety valve diminishing physiological responses to adverse circumstances^17,18^, we believe aggression and other forms of acting out can serve as a stress regulating function and thus mitigate increases in physiologic dysregulation. In sum, among adolescent youth growing up in high risk environments, where the associated stress may impair higher levels of cognitive regulation of emotion^67–69^, aggression may be an effective form of stress regulation. Aggression and bullying may have direct effects on stress regulation, or alternatively, because evidence suggests that they can confer resources and elevated status^24–26^, this increased access to both material and social resources can mitigate the effects of risk exposure.

The protective effect of externalizing behaviors is evident for males but not females. We posit a few possibilities for this sex difference. The first being greater social sanctions against aggression and violent behavior for girls^46,70^. These social norms might lead to greater recrimination, rejection, and punishment, effectively cancelling out any benefit of aggresion. Another possibility for the gender difference is that girls may have different pathways for coping with stress^53^. In particular, Taylor has posited that females have an increased likelihood of affiliating and mobilizing social support in conditions of stress. Research on adolescents have supported these premises that girls have more informal sources of support and are more likely to rely on these sources when in need^71^. A final important consideration for the difference is opportunities for emotion expression and processing. Because dampened emotional responses can lead to higher allostatic load, especially in the context of adversity^72^, differences in the extent to which females and males are allowed to express emotions may explain the gender difference. In particular, the expression of emotions such as sadness, fear, and embarrassment are evaluated more negatively in males^73^. Importantly, even when expressed they are less likely to be reassured and comforted as compared to females^74^. In sum, it is likely that the differences in patterns of stress regulation and coping strategies between males and females, as well as interactions with the larger socialcultural environment, led to the sex differences found herein. Future research specifically testing these mechanisms are warranted.

A few important caveats of our study need to be mentioned. First, we did not manipulate cumulative risk exposure in our study; thus, direction of causality cannot be definitive and third variable explanations cannot be ruled out. While reverse causality is unlikely, confounding variables leading to spurious associations between CR and AL cannot be ruled out. At the same time, our *a priori* prediction of an interaction reduces the plausibility that one or more unobserved variables could be the reason for our findings, as such confounding would have to explain the *interaction* of externalizing behaviors and cumulative risk exposure on the developmental outcome. Moreover, it is plausible that individuals who are lower on AL (hence healthier and perhaps stronger) are more likely to exhibit higher levels of externalizing behaviors. We believe this is unlikely, however, since we see either no relation between AL and externalizing behaviors in our data, or a small *positive* association suggesting that more dysregulation is associated with higher levels of externalizing. Future studies which focus on intervening to improve AL or lower levels of externalizing behaviors, however, are needed to ascertain direction of causality. Another drawback is the lack of generalizability of our results since the population studied is from rural areas and predominantly white. Because race and ethnicity influence patterns of discipline and punishment for aggressive behaviors^75^, our results may be very different depending on the racial make-up of the sample. For example, it is possible that youth of color may not be afforded the benefits of externalizing behaviors, due to increases in both likelihood and severity of punishment^76^. Finally, while we hypothesized that the effect would be stronger for males than females, we did not investigate specific mechanisms for these differences. Future research replicating our finding across different populations, as well as testing underlying mechanisms, is needed.

Regardless of these limitations, the longitudinal design and objective measures of stress in the current study further our understanding of adolescent behavioral problems. The results are potentially intriguing in thinking about notions of psychopathology and the extent to which “pathological” behaviors may be serving important functions. However, by no means are we suggesting that aggressive adolescent behavior is morally or functionally equivalent to behavior typically viewed as normative. To the contrary, such adaptive developmental mechanisms are likely to lead to maladaptive outcomes either through increased likelihood of harming others or a mismatch resulting from overuse—for instance, in a non-risky situation^64^. Interventions to prevent and address externalizing behaviors are necessary. However, strategies for addressing these behaviors would likely be most effective if the functional purpose of these behaviors was taken into account^7^, and the contexts that give rise to them considered. Outright prohibition or punishment of externalizing behaviors may have unintended harmful consequences, and at least for some youth in certain situations, remove one of their few effective coping strategies. Instead, training and educating adolescents to broaden their coping repertoire by applying more positive means of acting with confidence, asserting oneself, or learning alternative strategies to handle high risk settings would likely lead to better outcomes. Along these lines, rather than simply targeting aggression among adolescents, strategies such as offering opportunities for various forms of exercise and athletic activities^77,78^ or programs such as Mindfulness Stress Based Reduction (which have been shown to reduce stress, improve self-regulation and better focus energy when engaging with environmental demands^79^) seem far more likely to be effective and beneficial.

## Supplementary information

Table 1
