# Supplementary material for: Externalizing Behaviors Buffer the Effects of Early Life Adversity on Physiologic Dysregulation
Source: Sci Rep. 2019 Sep 20;9:13623. doi: 10.1038/s41598-019-49461-x (PMC6754506; doi:10.1038/s41598-019-49461-x)
Supplement: Supplementary file 1 — Table 1 [file 41598_2019_49461_MOESM1_ESM.docx]

Supplementary Information

For

Externalizing Behaviors Buffer the Effects of Early Life Adversity on Physiologic Dysregulation

Stacey N. Doan, Nadya Dich, Thomas E. Fuller-Rowell, Gary W. Evans

Table 1

Descriptive Statistics on Individual Cumulative Risk Factors

| Measure | *M* | *SD* |
| --- | --- | --- |
| Crowding (no. of people/room)  Noise (Leq. DBA)  Housing problems (range: 0-2)  Family separation (range: 0 -8)  Family turmoil (range: 0-8)  Violence (range: 0- 5)  Income-to-needs ratio  Single parent status^a^  Maternal education less than high school | .61  63.11  .60  1.88  1.65  .18  1.66  -  - | .20  7.15  .30  1.38  1.39  .23  1.09  -  - |

Note. ^a^Single parent status and maternal education less than high school is coded as 0 = absent or 1 = present. The proportion of single parents and low maternal edcuation is .44 and .07 respectively.
